# Popliteal artery pseudoaneurysm caused by distal femoral osteochondroma in a 13-year-old boy

**DOI:** 10.1016/j.jvscit.2026.102402

**Published:** 2026-07-07

**Authors:** Pucheng Wang, Yusheng Lin, Shuquan Wu, Hongwei Yang

**Affiliations:** Department of Vascular Surgery, Shenzhen Second People’s Hospital, Shenzhen, China

**Keywords:** Popliteal artery pseudoaneurysm, Osteochondroma, Pediatric vascular injury, Primary arterial repair

## Abstract

A 13-year-old boy presented with 10-day left distal thigh swelling and pain after running. Examination revealed a 7.0 × 9.0 cm firm popliteal mass, absent popliteal pulse, and weak dorsalis pedis pulse. Computed tomography angiography demonstrated a 69 × 91 mm pseudoaneurysm with multiple osteochondromas. Open repair included sac evacuation, primary repair of a 1.0 cm arterial defect with interrupted 6-0 polypropylene sutures, and excision of the offending osteochondroma with burr smoothing. The dorsalis pedis pulse was restored immediately, and recovery was uneventful. Osteochondroma is a rare cause of popliteal pseudoaneurysm in adolescents; however, open repair with simultaneous bony excision is safe and effective.

## Case report

This report was conducted in accordance with the Declaration of Helsinki; institutional review board approval was waived, and informed consent for publication was obtained. A 13-year-old boy was admitted with a 10-day history of swelling and pain in the left distal thigh. The symptoms began after a running test during physical education and persisted despite rest. He denied fever, skin redness, or preceding trauma. On physical examination, a 7.0 × 9.0 cm firm, immobile mass was palpable in the posteromedial aspect of the left distal thigh. The overlying skin was intact without erythema or warmth. The left knee was held in 120° of flexion with stiffness. The left popliteal pulse was absent; the dorsalis pedis pulse was weak, and the posterior tibial pulse was palpable, without calf swelling.

**Fig 1:**
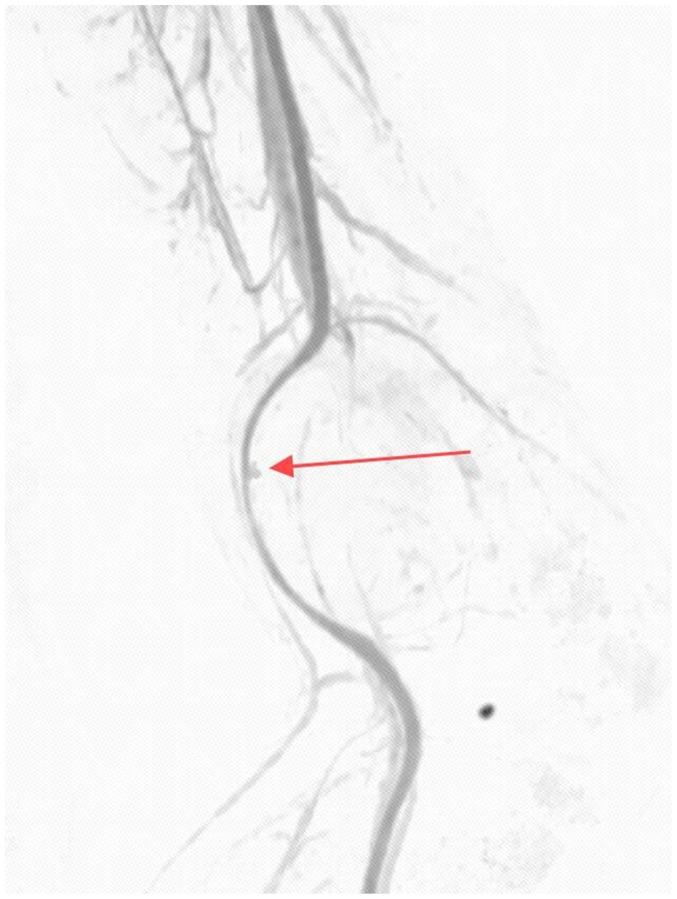
Contrast-enhanced computed tomography angiography of the left lower extremity demonstrating the popliteal artery pseudoaneurysm. The *red arrow* indicates the communication between the pseudoaneurysm sac and the popliteal artery.

**Fig 2:**
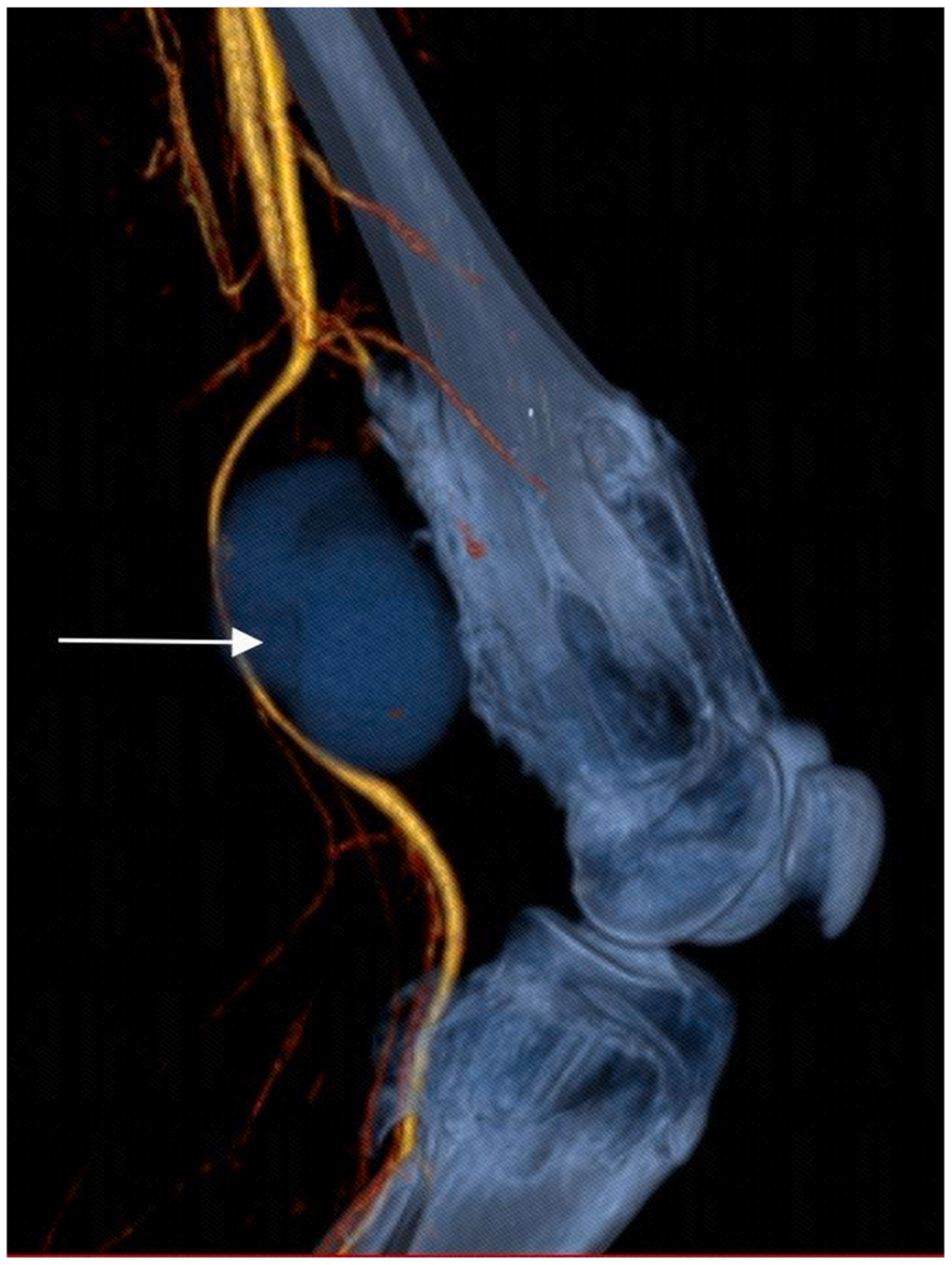
Three-dimensional volume-rendered computed tomography angiography reconstruction of the left knee. The *white arrow* indicates the popliteal artery pseudoaneurysm (*blue mass*) adjacent to the distal femoral metaphysis.

**Fig 3:**
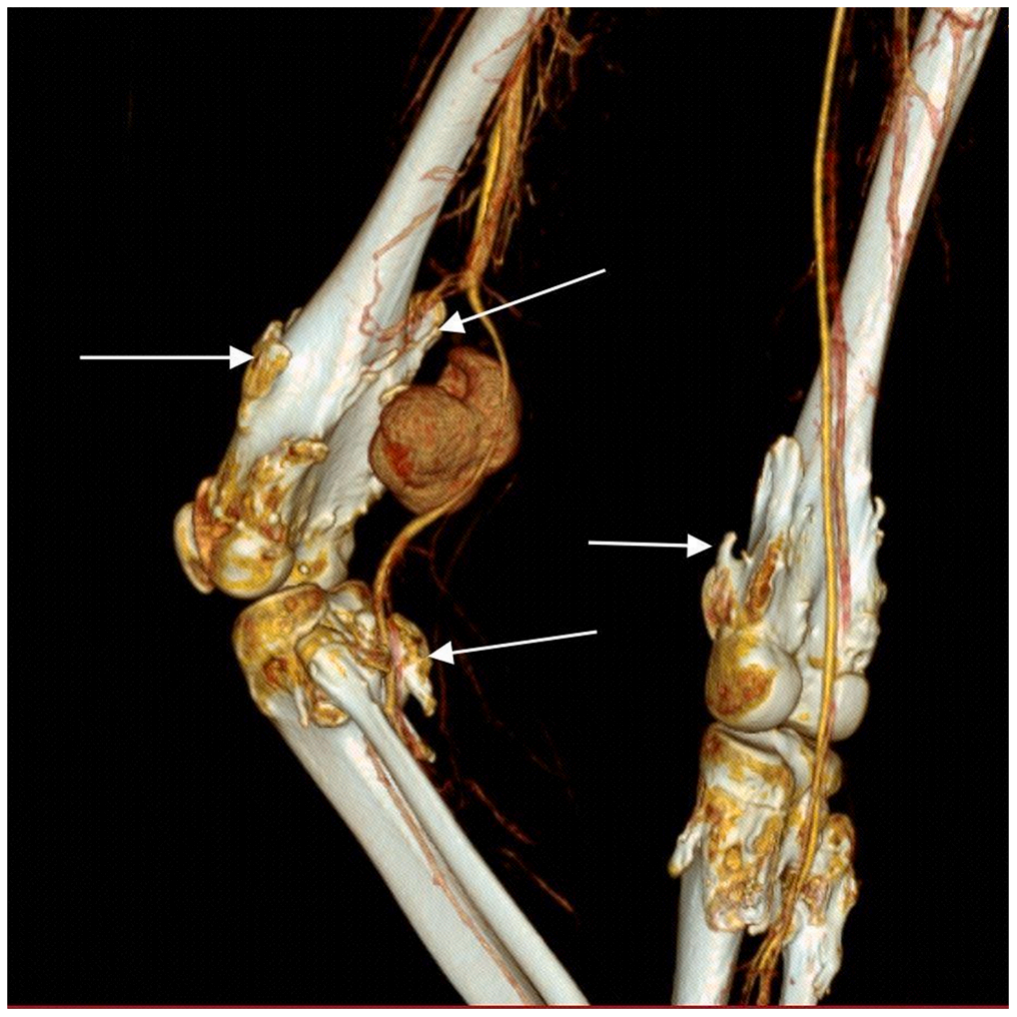
Three-dimensional volume-rendered computed tomography angiography reconstruction of bilateral lower extremities demonstrating multiple bony excrescences at the knee and ankle joints (*white arrows*), consistent with multiple osteochondromas. The left knee shows the associated popliteal artery pseudoaneurysm.

Contrast-enhanced computed tomography angiography (CTA) of the left lower extremity revealed a 69 × 91 mm mass in the left popliteal fossa with partial contrast opacification and a visible connection to the popliteal artery, consistent with a pseudoaneurysm (Figs 1 and 2). Incidentally, multiple bony excrescences were noted at bilateral knee and ankle joints, suggestive of multiple osteochondromas (Fig 3). The patient was admitted to the vascular surgery service with a diagnosis of left popliteal artery pseudoaneurysm and left lower-extremity osteochondroma.

**Fig 4:**
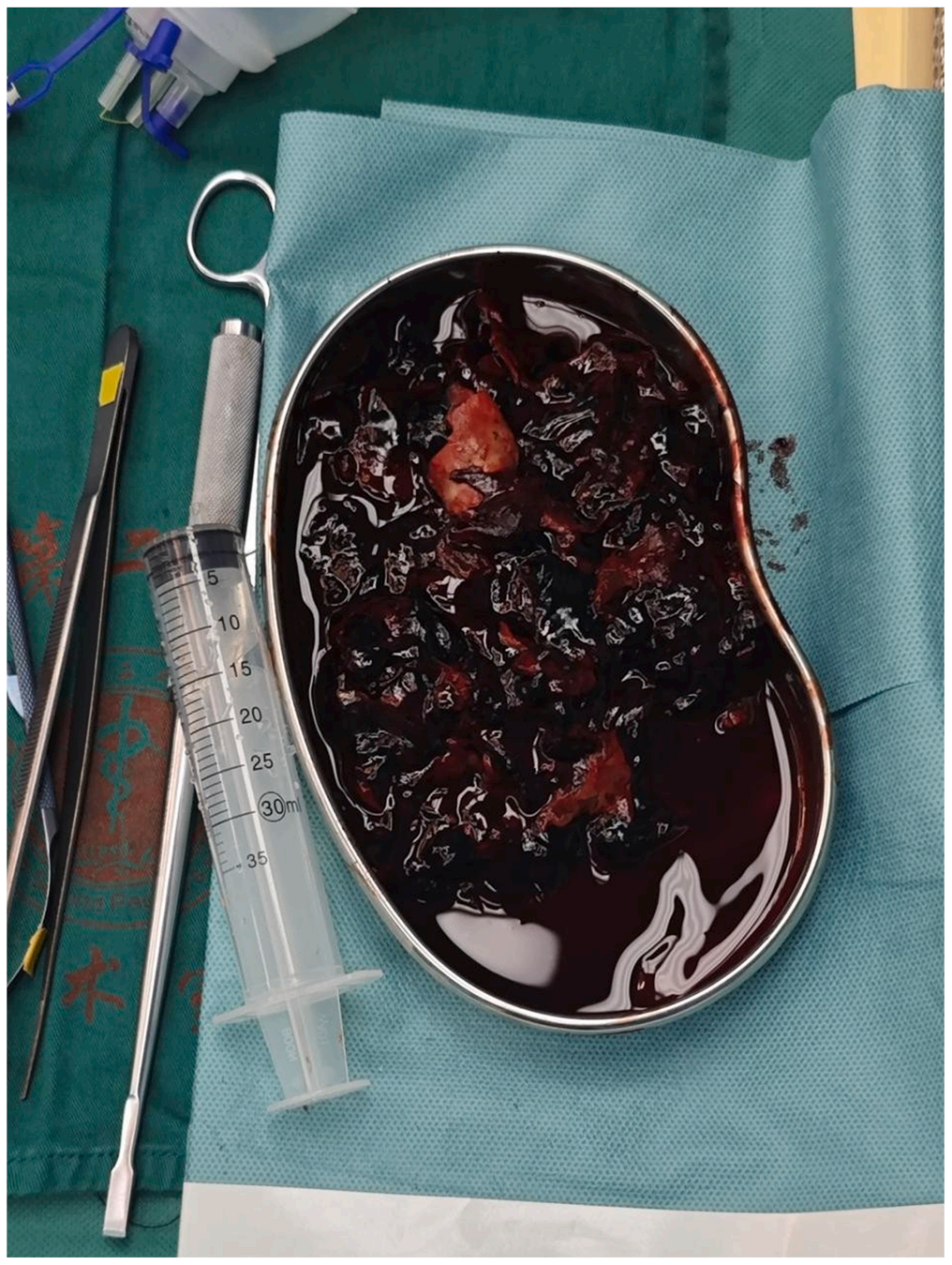
Evacuated hematoma and organized thrombi from the pseudoaneurysm sac.

**Fig 5:**
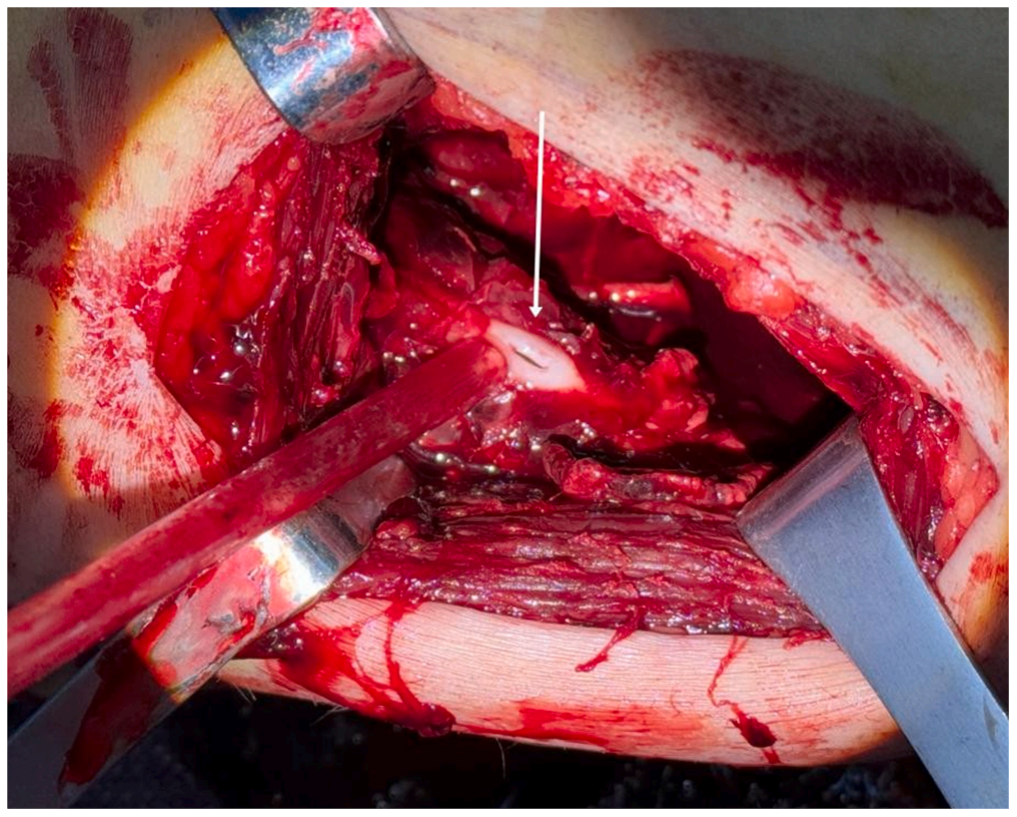
Intraoperative view demonstrating a 1.0 cm longitudinal tear in the posterior wall of the popliteal artery (*white arrow*) within the pseudoaneurysm sac after hematoma evacuation.

The patient underwent open repair under general anesthesia. Heparin was given before inflation. He was positioned supine, the left hip abducted and knee flexed. A pneumatic tourniquet was applied to the left proximal thigh. A 10 cm longitudinal incision was made along the medial aspect of the left distal thigh near the popliteal fossa. After incision of the skin, subcutaneous tissue, and deep fascia, blunt dissection through the vastus medialis was performed. A 7.0 × 10.0 cm cystic mass was identified posterior to the vastus medialis, containing dark bloody fluid and extensive clots. After evacuation of the hematoma (Fig 4), a 1.0 cm longitudinal tear was visualized in the popliteal artery posterior wall (Fig 5); transverse closure with interrupted 6-0 polypropylene sutures was performed. Posterior to the femur, a 1.0 × 2.0 cm bony spur was palpated, consistent with an osteochondroma. After release of the tourniquet, no active bleeding was observed, and a strong distal pulse was palpable. The osteochondroma was excised and the femoral cortex smoothed with a burr to eliminate the sharp edge and prevent recurrent injury. No soft tissue was interposed. The sac wall was preserved; the cavity was irrigated. The incision was closed over a drain. The procedure was completed without complications. Estimated intraoperative blood loss was 20 mL.

Histopathologic examination of the excised arterial wall specimen confirmed the diagnosis of pseudoaneurysm. Postoperatively, antiplatelet therapy was omitted. Dorsalis pedis and posterior tibial pulses remained strong. Twenty milliliters serosanguinous fluid drained. Predischarge duplex ultrasound examination confirmed patent popliteal artery flow without evidence of stenosis, thrombus, or recurrent pseudoaneurysm. The patient was discharged home in stable condition on postoperative day 7.

## Discussion

Popliteal artery pseudoaneurysms in children are rare and typically result from iatrogenic injury, blunt trauma, or penetrating trauma.^1^^,^^2^ Delayed presentation after minor trauma or mechanical irritation from adjacent bony structures has also been described.^3^, ^4^, ^5^ Spontaneous pseudoaneurysms caused by osteochondromas are exceedingly uncommon. Osteochondromas are the most common benign bone tumors in children and adolescents, arising from mutations in exostosin-1 or exostosin-2 genes; although vascular complications are rare, growth near neurovascular structures can cause compression, arterial erosion, or perforation.^6^ Miri et al^6^ recently reported a series of popliteal artery pseudoaneurysms secondary to osteochondromas, highlighting the potential for serious vascular injury when these lesions are located in the popliteal fossa.

The clinical presentation of popliteal artery pseudoaneurysm includes local pain, swelling, a pulsatile mass, arterial bruit, and neurocompressive symptoms.^7^ In the present case, the patient presented with a large mass, knee stiffness, and signs of distal ischemia manifested by absent popliteal and weak dorsalis pedis pulses. Compression of adjacent veins may lead to deep vein thrombosis.^8^ The absence of a pulsatile mass may be attributed to the large hematoma and thrombus within the sac. Duplex ultrasound examination is the preferred initial diagnostic modality, offering high sensitivity and specificity.^9^^,^^10^ However, it may be limited by patient discomfort or anatomic constraints in the acute setting.^11^ CTA provides rapid, noninvasive three-dimensional vascular imaging with excellent diagnostic accuracy,^12^ which is valuable particularly for preoperative planning in the popliteal fossa. In this case, CTA was instrumental in delineating the pseudoaneurysm and identifying the associated osteochondromas.

The optimal management of popliteal artery pseudoaneurysms in children remains undefined. Current options include conservative management, endovascular therapy, and open surgical repair.^13^ Ultrasound-guided thrombin injection has been described as a safe, minimally invasive option for femoral pseudoaneurysms in pediatric patients, with complication rates below 4%.^14^, ^15^, ^16^ However, this approach is generally reserved for small, hemodynamically stable, iatrogenic pseudoaneurysms without active extravasation. Coil embolization may be complicated by coil migration, particularly across a mobile joint, and covered stent placement risks stent fracture, migration, and interference with longitudinal bone growth in skeletally immature patients.^17^^,^^18^ Hemodynamic instability or large ruptured pseudoaneurysms often necessitate open surgical intervention.^19^

Open surgical repair was elected on the basis of four key considerations. A bypass strategy using autologous saphenous vein was planned if primary repair failed. First, the pseudoaneurysm was large (>3 cm) with mass effect, causing pain, immobility, and diminished perfusion, necessitating prompt reconstruction. Second, the deep popliteal location rendered endovascular strategies less attractive due to coil migration risk and poor stent graft durability across a flexion axis. Third, the patient was skeletally immature; implantation of a metallic stent or endograft could result in a fixed luminal diameter incompatible with future arterial growth, potentially leading to restenosis or occlusion, whereas primary repair preserves the native artery and its growth potential.^18^ Fourth, and most importantly, the mechanical irritation from the osteochondroma represented a persistent pathological stimulus; failure to remove the bony spur would leave the patient at ongoing risk for recurrent arterial perforation and pseudoaneurysm formation.^6^ After excision and smoothing, no muscle interposition was placed; however, this may be considered for high-risk patients.

To the authors' knowledge, popliteal artery pseudoaneurysm caused by femoral osteochondroma is extremely rare. Osteochondroma should be considered before CTA in adolescents with popliteal mass and bony protuberance, as it is visible on imaging. Open repair with primary suture and excision addresses vascular injury and its cause. Primary repair without prosthetic interposition is feasible for focal defects, advantageous in pediatric patients. Asymptomatic lesions should be monitored; excision is indicated for those near neurovascular structures, with sharp morphology, or progressive growth. Follow-up every 6 to 12 months is recommended. Malignant transformation is seen in 1% of solitary osteochondromas and in 3% to 5% of patients with hereditary multiple exostoses.^20^

This report is limited by its single patient, retrospective nature, and the absence of long-term follow-up data. Further studies are needed to establish standardized management and surveillance for popliteal artery pseudoaneurysms secondary to osteochondromas.

## Conclusions

This case demonstrates that distal femoral osteochondroma can cause popliteal artery pseudoaneurysm in adolescents through direct mechanical erosion. Open surgical repair with primary arterial reconstruction and concomitant removal of the osteochondroma is a safe and effective strategy that addresses both the acute vascular injury and the underlying etiology, while preserving the growth potential of the native artery.

## Funding

None.

## Patient consent

Written informed consent for publication of this case report and the accompanying images was obtained from the patient and his guardians.

## Data availability

All data supporting this case report are available from the corresponding author upon reasonable request.

## Disclosures

None.
